# Clinical study of auricular point seed burying combined with fire dragon pot moxibustion in perimenopausal women with insomnia

**DOI:** 10.1111/jog.15277

**Published:** 2022-05-04

**Authors:** Huan Feng, Aihong Pan, Guohua Zheng, Weihua Yu

**Affiliations:** ^1^ Nursing Department The Third Affiliated Hospital of Anhui Medical University (The First People's Hospital of Hefei) Hefei Anhui China; ^2^ Department of traditional Chinese Medicine The Third Affiliated Hospital of Anhui Medical University (The First People's Hospital of Hefei) Hefei Anhui China

**Keywords:** auricular point seed burying, fire dragon pot moxibustion, insomnia, perimenopausal women

## Abstract

**Objective:**

To intervene the insomnia symptoms of perimenopausal women by auricular point seed burying combined with fire dragon pot moxibustion, in order to improve the quality of sleep and life of the participants.

**Methods:**

Seventy female participants with perimenopausal insomnia who were treated with Chinese medicine techniques from January 2020 to October 2020 were randomly divided into a control group and an observation group, with 35 participants in each group. Participants in the control group were treated with the traditional Chinese medicine nursing intervention of burying seeds at auricular points. And participants in the observation group were additionally treated with fire dragon pot moxibustion. After 10 weeks of intervention, the Pittsburgh Sleepiness Index (PSQI), self‐assessment scores of anxiety (SAS) and depression (SDS), and treatment efficacy of the two groups were compared, respectively.

**Results:**

Before the intervention, there was no statistically significant difference in general information, sleep index scores, SAS, SDS scores between the two groups (*p* > 0.05). After the intervention, the SAS, SDS, and PSQI scores were significantly lower than the control group. Compared with the control group, the time to fall asleep was shorter and the duration of total sleep was longer in the observation group (*p* < 0.05). The treatment efficacy was better in the observation group (*p* < 0.05).

**Conclusion:**

Auricular point seed burying combined with fire dragon pot moxibustion therapy can be more effect than auricular point seed burying alone in treating perimenopausal women with insomnia.

## Introduction

Female menopausal participants often experience physical and psychological symptoms such as anxiety, depression, and sleep disturbances due to the weakening of the ovaries and hormonal changes in the body, with insomnia being the most typical symptom. Studies have shown that insomnia occurs in 75%–81% of perimenopausal women^1^ and is 2.4 times more common than in premenopausal participants.^2^ The main symptoms are difficulty falling asleep, early awakening and easy waking, which may be accompanied by anxiety, irritability, and sweating. Long‐term insomnia will lead to impairment of multisystem function and endanger the physical and mental health of perimenopausal women.^3^ The treatment of insomnia during menopause in western medicine is mainly based on pharmacological treatment, but it is controversial whether it is suitable for long‐term clinical application due to drug dependency and other side effects. Nonpharmacological traditional therapy in Traditional Chinese Medicine is more acceptable to participants because of its efficacy, nonchemical, and nontoxic side effects. Furthermore, it has been used in the clinical application of insomnia in perimenopausal participants with good efficacy in recent years.^4^ In this study, we used auricular seeds burying combined with fire dragon pot moxibustion as an appropriate Chinese medicine technique to treat perimenopausal women with insomnia, hoping to improve the life quality of perimenopausal women with insomnia.

## Materials and Methods

### Consent for publication

Written informed consent was obtained from the patient for publication of this case report and any accompanying images. A copy of the written consent is available for review by the journal editor.

### Subjects

Seventy female participants with perimenopausal insomnia who were treated with appropriate Chinese medicine techniques in a tertiary hospital in Hefei from January 2020 to October 2020 were selected and randomly divided into an observation group and a control group, with 35 participants in each group. Inclusion criteria: (1) all participants met the diagnosis of perimenopausal insomnia as described in the Chinese Guidelines for the Diagnosis and Treatment of Adult Insomnia (2017 Edition).^5^ (2) Pittsburgh Sleep Quality Index (PSQI) >7. (3) Insomnia at least three times per week and lasting for more than 1 month. (4) Onset around menopause with menstrual disorders. (5) No contraindications to auricular seed burial and fire dragon pot moxibustion treatment. (6) Informed consent.

Exclusion criteria: (1) those with a duration of less than 1 week or those who have received other relevant treatment within the last month that affects the observation of efficacy. (2) Secondary insomnia due to physical illness or psychological disorders. (3) Participants with severe comorbidities and psychiatric disorders. (4) Those who refuse to cooperate with treatment. (5) Those who use other methods of treatment during the treatment period, which affects the judgment of efficacy. Discontinuation criteria: (1) participants who are unable to complete the treatment cycle. (2) Those with serious adverse events. This clinical trial has been registered in the ClinicalTrials.gov (ID: NCT05234814) approved by the International Committee of Medical Journal Editors (ICMJE).

### Treatments

The control group was given the Traditional Chinese Medicine auricular acupuncture point buried seeds therapy. The observation group was additionally given the fire dragon pot moxibustion therapy.

#### Ear acupuncture seed burial method

According to “Ear Acupuncture Therapy,”^6^ the main acupuncture points were Shenmen, subcortical, sympathetic, endocrine, kidney, heart, liver, and spleen (all acupuncture points were positioned according to the national standard: GB/T12346‐2006).

Participants took a sitting position. Operators used an alcohol cotton ball to disinfect the auricle and dried it with a dry cotton ball, used an acupuncture point probe to take acupuncture points, and determined the acupuncture points after asking the participant if they had the feeling of soreness, numbness, swelling, and pain, which is called “de qi” in Chinese medicine. Then, 75% alcohol cotton ball was applied again to disinfect the corresponding acupuncture points locally. Operators then used Wangbuliuxing seed ear patch acupuncture points. The participants were instructed to apply pressure 4–5 times a day, for 30–60 s per point, with more pressure before bedtime. The auricular seeds were changed once a week, alternating between the two ears, for a total of 10 weeks of treatment.

#### Fire dragon pot moxibustion treatment

The main acupuncture points are as follows: Fengchi, Jianjin, Dazhui, Feiyu, Xinyu, Ganyu, Piyu, Shenyu, etc (all acupuncture points are positioned according to the national standard: GB/T12346‐2006).

Before applying the pot, the operators assisted the participants to lie prone on the treatment bed, exposed the upper body (used the curtain to cover, paid attention to protect the participant's privacy), applied emollient oil, chose a large fire dragon pot, ignited the moxa column in the pot. The operators should meet “one touch, two measurement, three observation”: that is, first, touch pot mouth has no rupture; second, measure whether pot mouth temperature is not too high; and finally test whether moxa column burning heating is uniform, heating is normal. Then, the operators held the pot with both hands and operated on the skin of the participant's neck, shoulder, and back. The main method of operation was to alternate between four types of techniques: moistening, scraping, plucking, and pointing. When applying the pot, the small fissure of the palm of the hand touched the skin firstly, and then gently slid the pot for the wetting method; after the participants had adapted to increase the downward pressure, the inner side of the pot mouth was used to push the muscles and fascia back and forth for the scraping method; the outer side of the pot mouth was used to push the muscles and fascia back and forth for the cupping method; the sharp part of the pot mouth was used to rub and pressed hard on a certain acupuncture point for the point method. First, operators used the wetting method to relax the muscles, then used the scraping method to loosen the fascia, then used the plucking method to stretch the muscles, and finally used the point method to stimulate the acupuncture points. The operation according to the temperature inside the pot appropriate adjustment boundary, to the skin red, sweating as the degree, the appearance of gua sha point that stop operation. During the operation, the participant's mental changes were constantly evaluated, and timely communication with the participants and observation of any adverse reactions such as erythema and blisters were made. After the treatment, participants were asked to pay attention to keep warm, avoid showering and contacting cold water within 4 h of treatment, and avoid eating cold food. The participants were treated once a week for 10 weeks.

### Observation indicators

The primary study outcome was the score of Pittsburgh Sleepiness Quantifier Inventory (PSQI), the secondary endpoints included the anxiety self‐assessment scale (SAS) and depression self‐assessment scale (SDS) scores, the time to fall asleep, the total sleep duration, and the safety indicators. We have described these in Section 2 according to your advice.

#### Pittsburgh Sleepiness Quantifier Inventory

The PSQI was compared between the two groups of patients before and after the intervention. The scale was developed by Buysse et al. to evaluate the sleep quality of the investigated subjects in the past month.^7^ Liu Xianchen et al.^8^ converted it into a Chinese version and conducted a reliability test. A PSQI score <7 is considered good sleep quantity and ≥7 is poor sleep quality, and the higher the patient score, the worse the sleep quality. In this study, the PSQI was measured by a professionally trained caregiver (fixed staff) before the intervention (first visit) and 1 week after the intervention session, respectively.

#### Anxiety and depression

The SAS and SDS scores were compared between the two groups before and after the intervention. In this study, the SAS and SDS developed by Zung were used. The SAS criteria: mild anxiety 50 to 59, moderate anxiety 60 to 69, and severe anxiety ≥70. SDS criteria: mild depression 53 to 62, moderate as 63 to 72, and severe anxiety ≥73.^9^ In this study, patients were measured by a professionally trained caregiver (fixed staff) before the intervention (first visit) and 1 week after the intervention session, respectively.

#### Efficacy criteria

According to the Clinical Research Guidelines for New Chinese Medicines for Insomnia^10^ and the Diagnostic Criteria for Chinese Medical Conditions,^11^ the efficacy diagnostic criteria are as follows: (1) cured, symptoms can be significantly improved, normal sleep state can be restored, and nighttime sleep time ≥6 h; (2) significant, insomnia symptoms are significantly improved, sleep quality increases, and nighttime sleep time increases ≥3 h; (3) effective, symptoms can be relieved or reduced, and nighttime sleep time increases <3 h; (4) ineffective, symptoms do not show any improvement.

#### Safety indicators

Observe whether erythema and blisters are appearing at the fire dragon pot walking site and whether there are allergic and other adverse reactions in the two groups of patients. Moreover, find whether there are any adverse reactions such as allergy to adhesive tape, skin breakage, and infection at the site of buried seeds in the ear acupuncture points of patients in both groups.

### Statistical methods

An initial sample size of 68 was calculated by using PASS 15.0.5 software based on *α* = 0.05, *β* = 0.20 (two‐tailed test), *δ* = 1.4, *σ* = 1.1 (according to our previous clinical observation, the Pittsburgh Sleepiness Index decreased 7.1 ± 2.2 in patients treating with Traditional Chinese Medicine auricular acupuncture point buried seeds plus the fire dragon pot moxibustion therapy, while the Pittsburgh Sleepiness Index decreased 5.7 ± 1.1 in patients treating with Traditional Chinese Medicine auricular acupuncture point buried seeds therapy only.), and power = 90%. And to consider a possible 5%–10% missing data, the final sample size was determined as 70 patients. SPSS 24.0 statistical software was used for data analysis. The *χ* ^2^ test was used for the categorical data when comparing two groups. The measurement data conforming to normal distribution were shown as (X ± s), and the *t* test was used for comparison. *α* = 0.05 was used as the test level and *p* < 0.05 was considered a statistically significant difference.

## Results

**FIGURE 1:**
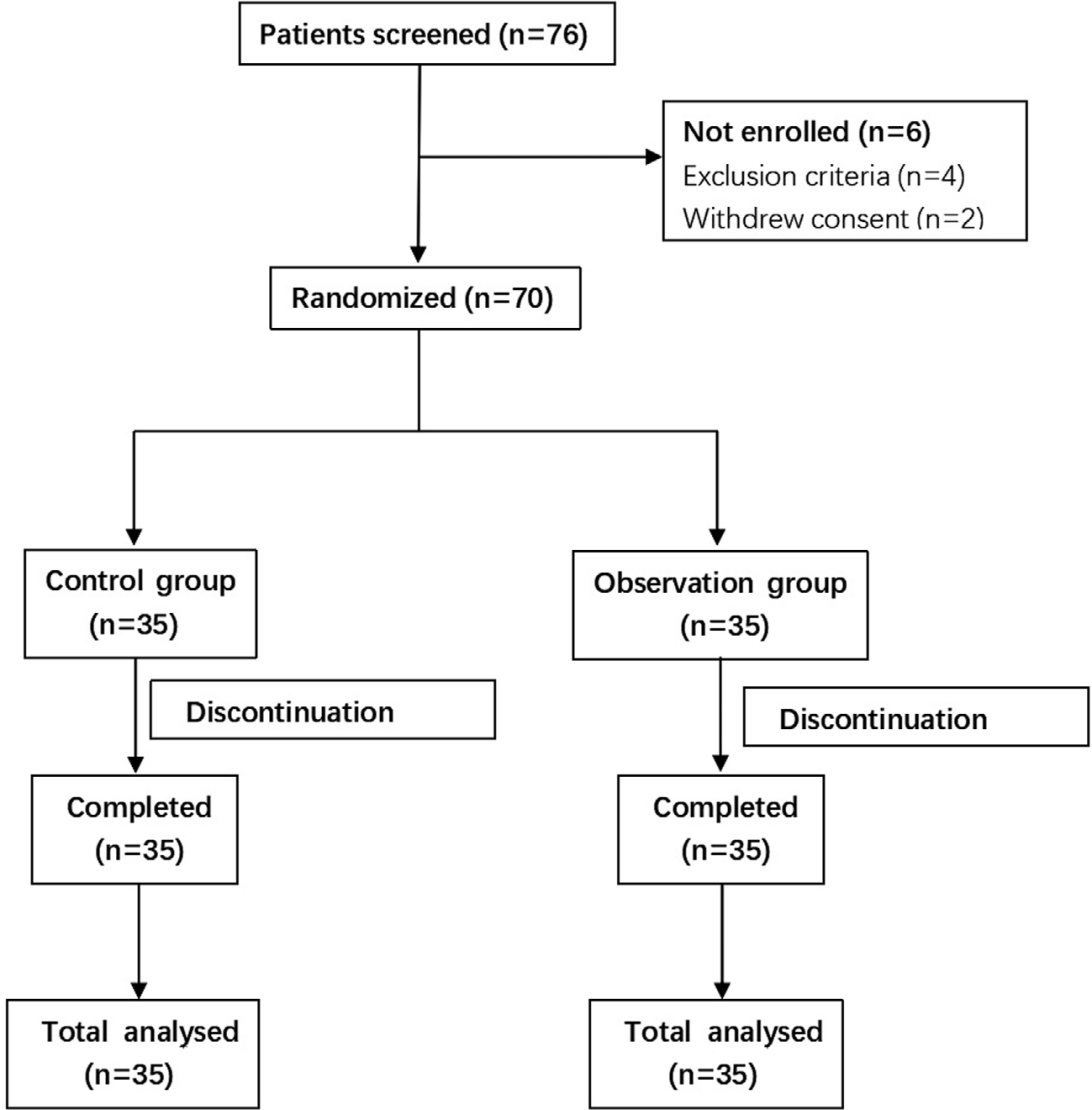
Flow diagram of the participants in this study

A total of 76 patients were screened, and 6 patients were excluded because they did not meet the exclusion criteria or they withdrew consent. During the study, none of the patients discontinued the study (Figure 1).

There was no statistically significant difference between the two groups in terms of age, culture, marital status, and other general information (*p* > 0.05) (Table 1).

**TABLE 1:** Comparison of general information between two groups of participants

		Observation group	Control group	*X* ^2^/*t*	*p*
Age		50.91 ± 4.45	51.03 ± 3.94	−0.114	0.910
Education	Below junior high school	4	6	0.467	0.792
	Junior high school or above, college or below	16	15		
	College and above	15	17		
Marital status	Married	33	34	0.348	0.555
	Divorced/widowed	2	1		
Occupation	Government worker	10	7	1.269	0.736
	Company worker	13	12		
	Retirement	3	5		
	Unemployed	9	11		

### Anxiety and depression status

Before treatment, there was no statistically significant difference between the SAS scores and SDS scores of the two groups (*p* > 0.05). After the intervention, the SAS scores and SDS scores of the observation group were significantly lower compared with the control group (*p* < 0.05), as shown in Table 2.

**TABLE 2:** Comparison of treatment SAS and SDS scores between two groups of participants after intervention

Group	SAS scores		SDS scores	
	Before	After	Before	After
Observation group	57.57 ± 6.54	48.07 ± 4.49	57.83 ± 6.74	47.97 ± 4.48
Control group	57.29 ± 7.73	52.57 ± 7.52	57.76 ± 7.68	52.44 ± 7.29
*t*	0.167	3.038	0.40	3.068
*p*	0.868	0.004	0.968	0.003

Abbreviations: SAS, self‐assessment scores of anxiety; SDS, self‐assessment scores of depression.

### Comparison of Pittsburgh sleepiness index before and after treatment between the two groups

Before treatment, there was no statistically significant difference in PSQI scores, time to sleep and total sleep time between the two groups (*p* > 0.05). After the intervention, PSQI scores and time to sleep were significantly lower and total sleep time was significantly higher in the observation group compared with the control group (*p* < 0.05), as shown in Table 3.

**TABLE 3:** Comparison of Pittsburgh sleepiness index (PSQI) before and after treatment in both groups

Group	PSQI scores		Time to fall asleep (min)		Total sleep duration (min)	
	Before	After	Before	After	Before	After
Observation group	11.80 ± 3.05	4.29 ± 1.36	75.43 ± 41.87	19.71 ± 9.99	234.0 ± 55.54	343.14 ± 40.05
Control group	12.06 ± 2.43	6.11 ± 1.55	76.29 ± 42.01	28.79 ± 9.68	235.5 ± 55.57	312.57 ± 37.44
*t*	5.224		−2.808		3.298	
*p*	0.000		0.007		0.002	

### Comparison of the treatment effect between the two groups of participants

The treatment effect of participants in the observation group was better than that in the control group (*χ* ^2^ = 11.882, *p* = 0.008), and the difference was statistically significant (Table 4).

**TABLE 4:** Comparison of treatment effects between the two groups of participants after intervention (n, %)

Effect	Observation group	Control group	X^2^	P
Cured	9 (25.71%)	3 (8.57%)		
Significant	1 (34.28%)	6 (17.14%)		
Effective	14 (40%)	21 (60.0%)	11.4	0.007
Ineffective	0	5 (14.28%)		

### Safety indicators

No adverse reactions such as erythema, blistering, adhesive tape allergy, skin breakdown, or infection were observed in all participants in this study.

## Discussion

Modern medical research on the pathogenesis of insomnia in perimenopausal women is inconsistent, and the current unified view is that insomnia in perimenopausal women is related to endocrine changes and psychosocial factors in this stage.^12^ According to Chinese medicine, the cause of perimenopausal insomnia is mainly in the heart, and the pathogenesis is based on kidney deficiency, and is closely related to the heart, liver, and spleen. The main manifestations of perimenopausal insomnia are heart and kidney disconnection, liver and kidney deficiency, heart and spleen deficiency.^13^ Long‐term insomnia can cause physical and psychological disorders in perimenopausal women, which seriously affects the physical and psychological health of perimenopausal women.

Western medicine often uses sedative sleeping drugs in the treatment of insomnia in perimenopausal women, which have better effects but have more side effects, and whether they are suitable for long‐term application is still controversial. In clinical practice, nonpharmacological traditional therapies in Traditional Chinese Medicine are easy to be accepted by patients because of their simplicity, safety, and effectiveness, and have achieved significant clinical efficacy in the treatment of perimenopausal insomnia patients in recent years.^14^, ^15^ The Ling Shu—Mouth Question^16^ indicated that the ear is closely related to the five internal organs. As a characteristic external treatment method of Traditional Chinese Medicine, seed embedding in ear points stimulates the corresponding ear points to unblock the meridians, and regulate and balance the internal organs for the purpose of treating diseases. As a nondrug therapy in Traditional Chinese Medicine, the auricular seed burying method is easy to accept by patients because of its exact efficacy, no chemical and drug stimulation and drug toxic side effects, simple operation, safety and reliability, economy, and practicality. Furthermore, after the doctor or nurse selected and applied the acupuncture points, patients can press the treatment site by themselves under the guidance of medical personnel, and it is not restricted by environment and place. Several studies in recent years^14^, ^15^ have shown its efficacy in treating menopausal insomnia symptoms. The fire dragon pot is a special pot developed by Liu Weicheng based on comprehensive Tui Na, cupping, moxibustion and Gua Sha, with an irregular petal‐shaped mouth and a moxa pillar inside.^17^ The special design of the pot mouth allows for walking, scraping, and rubbing of acupuncture points, while three 3 cm diameter moxa pillars can be placed inside the pot. When lit, the moxa pillar becomes a firepot, which can drive away cold, remove dampness, and resolve blood stasis. Fire Dragon pot is a comprehensive Chinese medicine treatment tool that integrates Tui Na, Moxibustion, and Gua Sha. This treatment method can achieve the effect of regulating the internal organs, unblocking the meridians, activating blood, slipping joints, and warming the body. It has been applied in the study of lumbar disc herniation^17^ and insufficient menstrual flow^18^ and achieved remarkable results. Therefore, our study combined these two treatment and found that the combination of both interventions more effective than auricular point seed burying in treating perimenopausal female for insomnia symptoms.

The current study has some limitations. The intervention study was conducted in only one hospital and the sample size was small. It is hoped that in future studies, multicenter collaboration can be conducted to expand the sample size and further explore the application of auricular seed burial combined with fire dragon pot moxibustion therapy technique in perimenopausal women with insomnia.

In this study, the control group used a single auricular seed burial technique intervention and the observation group was treated with auricular seed burial combined with fire dragon pot moxibustion methods. The results showed that the PSQI, SAS, and SDS scores were significantly lower in the observation group than in the control group, and the patient treatment efficacy was significantly higher in the observation group than in the control group. The composite treatment method used in this study combined the strengths of both auricular seed burial and fire dragon pot moxibustion, making the treatment effects superimposed and effectively improving the sleep quality of perimenopausal female patients. This study pioneered a new nonpharmacological combination treatment method in Traditional Chinese Medicine, which provides a theoretical basis for the application of Traditional Chinese Medicine appropriate techniques in perimenopausal female patients with insomnia. It is worthy of further promotion.

## Conflict of interest

The authors declare that they have no competing interests.
